# The use of a neutral peptide aptamer scaffold to anchor BH3 peptides constitutes a viable approach to studying their function

**DOI:** 10.1038/cddis.2013.564

**Published:** 2014-01-30

**Authors:** L K J Stadler, D C Tomlinson, T Lee, M A Knowles, P Ko Ferrigno

**Affiliations:** 1 Section of Experimental Therapeutics, Leeds LS9 7TF, UK; 2 Medical Research Council Laboratory of Molecular Biology, Cambridge CB2 0QH, UK; 3 Section of Experimental Oncology, Leeds Institute of Molecular Medicine, St James's University Hospital, Beckett Street, Leeds LS9 7TF, UK

**Keywords:** peptide aptamer, BH3 domains, Bcl-2, apoptosis

## Abstract

The B-cell CLL/lymphoma-2 (Bcl-2) family of proteins are important regulators of the intrinsic pathway of apoptosis, and their interactions, driven by Bcl-2 homology (BH) domains, are of great interest in cancer research. Particularly, the BH3 domain is of clinical relevance, as it promotes apoptosis through activation of Bcl-2-associated x protein (Bax) and Bcl-2 antagonist killer (Bak), as well as by antagonising the anti-apoptotic Bcl-2 family members. Although investigated extensively *in vitro*, the study of the BH3 domain alone inside cells is more problematic because of diminished secondary structure of the unconstrained peptide and a lack of stability. In this study, we report the successful use of a novel peptide aptamer scaffold – Stefin A quadruple mutant – to anchor and present the BH3 domains from Bcl-2-interacting mediator of cell death (Bim), p53 upregulated modulator of apoptosis (Puma), Bcl-2-associated death promoter (Bad) and Noxa, and demonstrate its usefulness in the study of the BH3 domains *in vivo*. When expressed intracellularly, anchored BH3 peptides exhibit much the same binding specificities previously established *in vitro*, however, we find that, at endogenous expression levels, Bcl-2 does not bind to any of the anchored BH3 domains tested. Nonetheless, when expressed inside cells the anchored PUMA and Bim BH3 *α*-helices powerfully induce cell death in the absence of efficient targeting to the mitochondrial membrane, whereas the Noxa helix requires a membrane insertion domain in order to kill Mcl-1-dependent myeloma cells. Finally, the binding of the Bim BH3 peptide to Bax was the only interaction with a pro-apoptotic effector protein observed in this study.

Programmed cell death is a tightly controlled process encompassing a wide range of regulatory mechanisms. Permeabilisation of the mitochondrial membrane and the resulting release of cytochrome *c* into the cytoplasm is a central event in the induction of the intrinsic apoptotic pathway. The B-cell CLL/lymphoma-2 (Bcl-2) family of proteins is chiefly involved in the regulation of apoptosis at the mitochondrion.^1, 2, 3, 4, 5^ As well as apoptosis, more recent evidence has implicated the Bcl-2 family in the regulation of mitochondrial morphology and autophagy.^6, 7^ Structurally, the Bcl-2 proteins are characterised by four alpha helical Bcl-2 homology domains (BH1-4), at least one of which is present in each family member. In functional terms, the protein family is divided into three groups: the apoptotic effector proteins Bcl-2-associated x protein (Bax) and Bcl-2 antagonist killer (Bak; and possibly Bok), the anti-apoptotic proteins Bcl-2, Bcl-2-related gene long isoform (Bcl-xl), Bcl-w, Mcl-1 and Bcl-2-related gene A1 (A1), and finally the pro-apoptotic BH3-only proteins. In the last group, one can further distinguish between activators (Bcl-2-interacting mediator of cell death (Bim) and Bcl-2-interacting domain death agonist (Bid)) and sensitisers (e.g., p53 upregulated modulator of apoptosis (Puma), Bcl-2-associated death promoter (Bad), Noxa, Bcl-2-interacting killer (Bik) and more). In the event of cytotoxic stress, Bak and/or Bax oligomerise and produce pores in the mitochondrial outer membrane (MOM) thus initiating MOM permeabilisation (MOMP) and cytochrome *c* release.^3^ The mechanism of Bcl-2 family-regulated apoptosis is predominantly described by either the direct or indirect activation models, or a combination thereof (reviewed in Chipuk *et al.*^3^ and Shamas-Din *et al.*^5^).

The pro-apoptotic BH3-only proteins are diverse in structure and largely unrelated except for their BH3 domain, which represents the minimal unit of their function and is necessary for target protein binding and induction of apoptosis.^8, 9, 10, 11^ Binding of the BH3-only Bcl-2 family members to each of their anti-apoptotic counterparts occurs in a selective manner, which closely correlates with their apoptotic potency.^12, 13^ Bim, Puma and Bid have been found to bind to all five anti-apoptotic Bcl-2 proteins; when overexpressed in cells they potently induce cell death. Bad and Noxa, on the other hand, bind to complementary subsets of anti-apoptotic factors, with Bad interacting strongly with Bcl-2, Bcl-xl and Bcl-w and Noxa binding to Mcl-1 and A1.^11, 14^ Overexpression of Bad and Noxa individually failed to cause apoptosis in mouse embryonic fibroblasts.^12^ Furthermore, Bim and Bid (and maybe Puma) directly bind and activate Bax and Bak.^8, 15, 16, 17^

As aberrant regulation of apoptosis by the Bcl-2 family often lies at the heart of many diseases, the development of molecular tools, which help dissect the pathways involved, is of paramount importance. In particular, the role of the BH3 domain in isolation (i.e., outside the context of the full-length protein) as the mediator of the death signal is of significance to clinical research, as this interaction site has become an attractive drug target in the treatment of cancer.^18, 19^ The interactions of the BH3 peptides with the anti-apoptotic and pro-apoptotic-effector Bcl-2 proteins have been studied thoroughly *in vitro*, however, the BH3-only proteins are not easily express recombinantly,^12^ making it difficult to derive reliable conclusions from peptide-based interaction studies. Moreover, introducing peptides into cells is challenging and once inside peptides are susceptible to proteolytic degradation.^20, 21^ Consequently, rather less emphasis has been placed on the study of the BH3 peptides *in vivo*. A further caveat to the use of the BH3 peptides in isolation is their lack of secondary structure, which – in the absence of a structural constraint – precludes high-affinity interactions with their targets.^16, 22^

We have recently reported the design and validation of a series of new peptide aptamer scaffolds, culminating in Stefin A quadruple mutant Tracy (SQT), derived from the mammalian protease inhibitor Stefin A.^23^ Peptide aptamers represent a relatively new class of alternative binding proteins, and are defined as binding peptides constrained within a protein scaffold.^24^ As binding molecules they are superior to free peptides, which are susceptible to proteolytic cleavage in cells, and which – because of their flexible nature – tend to bind to protein surfaces non-specifically. In this study, we explore the use of a novel peptide aptamer scaffold to anchor and present four different BH3 domains as a means of investigating their functions when expressed in cells. We propose that such tools may in time allow a direct correlation between biochemical and intracellular studies of BH3 helix function.

## Results

### Structural analysis of the scaffold-constrained BH3 domains

**Figure 1:**
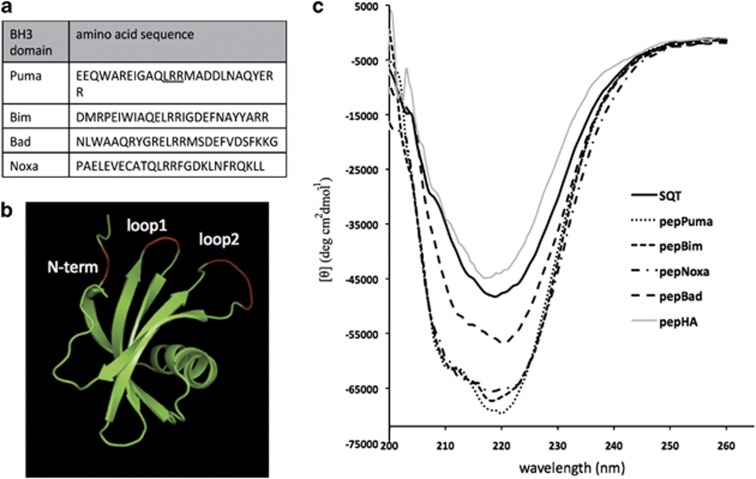
Structural analysis of the SQT scaffold presenting four different pro-apoptotic BH3 domains. (**a**) Amino-acid sequence of the four BH3 peptides used in this study. In the Puma sequence, the underlined amino acids are deleted in pepPumaDel. (**b**) Nuclear magnetic resonance (NMR) structure of Stefin A (adapted from pdb ref: 1DVC). The three sites for peptide presentation in the SQT peptide aptamer scaffold are highlighted in red. (**c**) The CD spectrum of SQT alone and with N-terminal insertions of the HA tag (a negative control not expected to adopt an alpha helical configuration) or with each BH3 peptide. The single inflection point around 218 nm indicates predominantly *β*-strands in the SQT and SQT-HA spectra. A shift in the inflection point to 220–222 nm, the deeper inflection of the spectrum and an additional shoulder signal the alpha helix formed by the BH3 peptides. SQT with Noxa BH3 and the HA peptide have been reported previously^23^

Based on previous studies,^12, 25^ 26-amino-acid stretches covering the BH3 region of Puma, Bim, Noxa and Bad, respectively (Figure 1a), were introduced into the N-terminal insertion site of the SQT peptide aptamer scaffold (Figure 1b), thus creating pepPuma, pepBim, pepNoxa and pepBad (collectively referred to as ‘pepBH3'). Circular dichroism (CD) spectroscopy was used to assess the secondary structure of the pepBH3 proteins (Figure 1c). The empty SQT scaffold contains predominantly *β*-strands, which is clearly reflected in its CD spectrum. Insertion of the BH3 domains altered the CD spectrum in a manner that is indicative of *α*-helices. When introducing the HA epitope tag into the same insertion site, the CD spectrum of SQT remains largely unchanged, suggesting that anchoring the BH3 peptides within a larger open reading frame (ORF) allows them to adopt a stable *α*-helical conformation.

### Functional analysis of pepBH3

**Figure 2:**
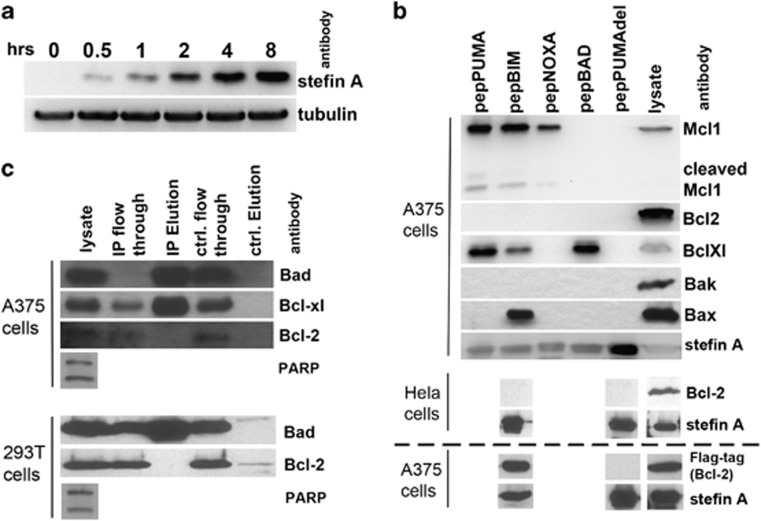
PepBH3 expression and identification of their binding partners. (**a**) Following induction, pepBH3 expression is detectable after 30 min. (**b**) Co-IP of pro- and anti-apoptotic Bcl-2 proteins with pepBH3 following their expression in A375 (top panel) and Hela cells (middle panel). There is no interaction between Bcl-2 and pepBim in A375 or Hela cells, however, when Bcl-2 is overexpressed (in A375) binding to pepBim is significant (bottom panel, underneath dashed line). (**c**) IP of endogenous full-length Bad from A375 cell lysate reveals binding of Bcl-xl, but not Bcl-2 (top panel). Equally in 293T cells, the Bad:Bcl-2 interaction does not occur (bottom panel). PARP cleavage indicates that apoptosis was induced successfully in these cells

To study these newly created pepBH3 proteins intracellularly, a retroviral system (ProteoTuner) was used to stably transduce A375 melanoma cells with the pepBH3-encoding ORFs. In this expression system, proteins are constitutively targeted to the proteasome until addition of a chemical stabiliser (Shield).^26^ To confirm the suitability of the ProteoTuner system, expression of SQT after addition of Shield (5 *μ*M) at numerous time points was examined (Figure 2a). SQT protein was detectable after 0.5 h and increased during the time course. Next, co-immunoprecipitation (co-IP) was used to investigate the ability of each pepBH3 protein to interact with other Bcl-2 protein family members inside cells (Figure 2b). PepPuma and pepBim co-IP with the anti-apoptotic proteins Bcl-xL and Mcl-1, whereas pepBad only co-IP with Bcl-xL and pepNoxa with Mcl-1, the latter as previously reported.^23^ The only interaction detectable between a pepBH3 and a pro-apoptotic effector protein was between pepBim and Bax. No *in vivo* binding to Bak was observed. A functionally inactive version of pepPuma (pepPumaDel), with a mutated BH3 domain^27^ where three amino acids, which are crucial to the protein–protein interaction, have been deleted (Figure 1a) did not bind to any of the Bcl-2 family members assayed. Surprisingly, none of the pepBH3 proteins showed intracellular interaction with Bcl-2, which was nonetheless present. We performed a co-IP experiment using an anti-Bad antibody, to test whether endogenous, full-length Bad was bound to endogenous Bcl-2 in staurosporine-treated A375 cells (Figure 2c). Although Bad successfully co-IP with Bcl-xL, no interaction with Bcl-2 was observed, consistent with the pepBad result (Figure 2b). In order to ascertain whether this surprising observation was specific to A375 cells, two separate experiments were carried out. First, Hela cells, transfected with pepBim and the negative control pepPumaDel, respectively, were submitted to a co-IP experiment (Figure 2b). Again no interaction between pepBim and endogenous Bcl-2 was detected. Second, the binding of endogenous full-length Bad to endogenous Bcl-2 was tested in the non-cancerous cell line human embryonic kidney 293T cells (Figure 2c). As for the A375 cells, an anti-Bad antibody column was used to establish whether Bcl-2 IP with Bad in apoptosing cells and again the result was negative.

As all the experiments so far had looked at endogenous levels of Bcl-2, we decided to test the interaction with pepBim when Bcl-2 was overexpressed. To that end, a mammalian expression vector carrying Flag-tagged Bcl-2 was transiently transfected into A375 cells carrying pepBim and pepPumaDel, respectively. Following transfection with Bcl-2 and induction of peptide aptamer expression, the cells were lysed (4 h after addition of 5 *μ*M Shield) and co-IP carried out using an anti-Stefin A antibody (Figure 2b, bottom panel). This time efficient binding of pepBim to Bcl-2 could be detected, while no interaction with pepPumaDel occurred.

**Figure 3:**
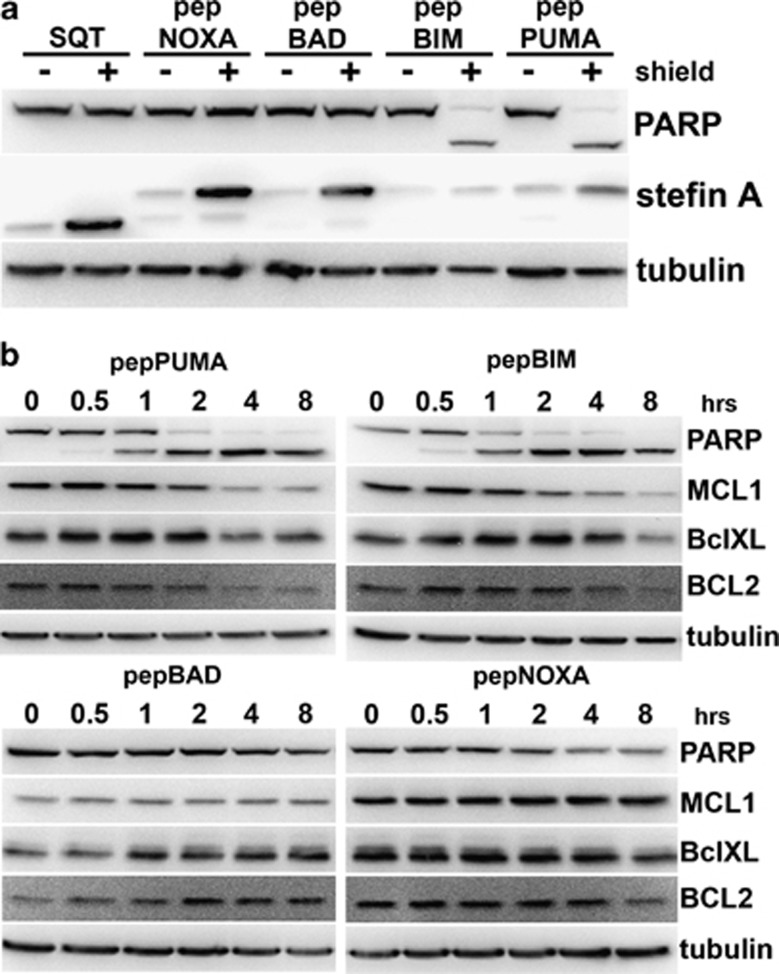
PARP cleavage in A375 cells expressing pepBH3 proteins. (**a**) PepBim/Puma expression results in cleavage of PARP. (**b**) Cleavage of PARP occurs as early as 30 min after PepBim/Puma expression is initiated and downregulation of anti-apoptotic proteins starts after 2–4 h

Next, cleavage of PARP was monitored to test whether pepBH3 proteins are capable of inducing apoptosis (Figure 3a). Expression of either pepPuma or pepBim, but not of pepBad or pepNoxa, results in efficient PARP cleavage. This is consistent with the ability of pepPuma and pepBim to bind to Bcl-xL and Mcl-1 (Figure 2b) and with the previously reported apoptotic potential of each of the full-length proteins.^12^ PARP cleavage is detectable within 30 min of induction with Shield in cells expressing pepPuma or pepBim, and a significant decrease in the levels of the anti-apoptotic proteins Mcl-1, Bcl-2 and Bcl-xL are observed at 4 h (Figure 3b).

### PepBim/Puma weakly localise to the mitochondria, but strongly induce apoptosis

**Figure 4:**
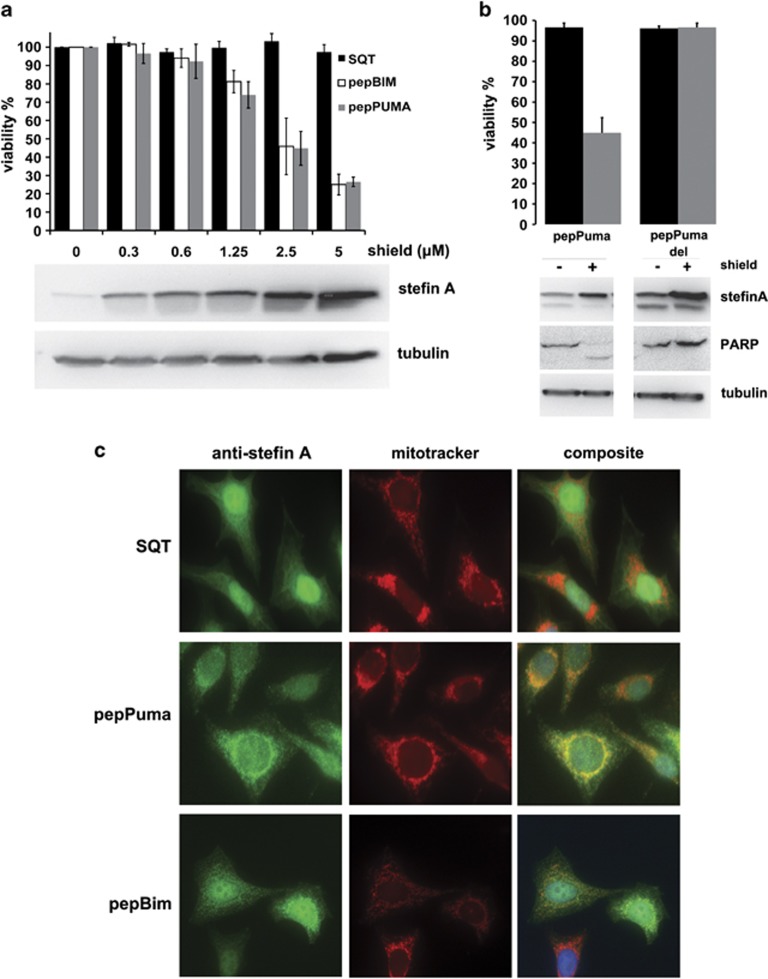
Cell viability and localisation of pepPuma/Bim in cells. (**a**) PepPuma/Bim expression in A375 cells reduces cell viability by approximately 80%. The western blot shows levels of SQT with increasing amounts of Shield (error bars are ±S.D.; *n*=3). (**b**) PepPumaDel does not cause cell death (error bars are ±S.D.; *n*=3). (**c**) Immunofluorescence of Hela cells expressing SQT reveals no specific localisation pattern. PepBim or pepPuma (green) colocalise with mitotracker (red) in some but not all cells. DAPI-stained nuclei are shown in blue

A cell viability assay following induction of pepBim/Puma expression in A375 cells with increasing amounts of Shield reveals that these peptide aptamers efficiently cause cell death. Induction of protein expression with 5 *μ*M shield leads to a reduction in cell viability by 80% (Figure 4a). PepPumaDel does not affect cell viability (Figure 4b). To study the cellular localisation of the peptide aptamers immunocytochemistry (ICC) was used to probe for pepBim, pepPuma and empty SQT in Hela cells expressing these peptide aptamers (Figure 4c). SQT is expressed at high levels in the cells and displays a diffuse staining pattern, distributed evenly across all cellular compartments. PepPuma and pepBim show increased localisation to areas rich in mitochondria, especially surrounding the nucleus, however, staining remains rather diffuse and exhibits variability across different cells. These observations are consistent with the fact that pepBim/Puma lack the C-terminal hydrophobic domains of the full-length proteins, which have previously been shown to be essential for mitochondrial localisation.^28, 29^ We conclude that the BH3 helix is sufficient for induction of apoptosis even in the absence of a mitochondrial-targeting sequence.

**Figure 5:**
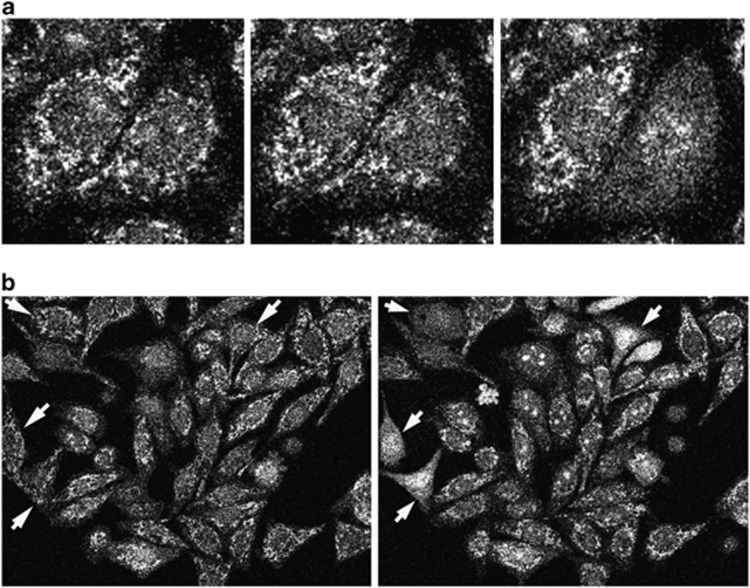
PepPuma expression results in cytochrome *c* release from the mitochondria. (**a**) Shown are three time points in the process of apoptosis. In the left-hand panel, both cells in the main field of view have their mitochondria intact. In the central panel, the right-hand cell is starting to undergo apoptosis with mitochondrial membrane permeabilisation leading to the GFP signal becoming more diffuse. In the right-hand panel, the right-hand cell has completely lost mitochondrial integrity and GFP–cytochrome *c* has translocated to the nucleus. (**b**) White arrows indicate cells before (left panel) and after (right panel) mitochondrial permeabilisation

In order to verify that the BH3 presenting peptide aptamers cause programmed cell death via initiation of cytochrome *c* release from the mitochondrion, a Hela cell line, which stably expresses a green fluorescent protein (GFP)–cytochrome *c* fusion protein, was used (Figure 5). In a healthy cell, the mitochondria are visible as distinct features under the confocal microscope (Figure 5a). Upon expression of pepPuma, cytochrome *c* is released efficiently starting in some cells from 30 to 60 min following induction. Visually this can be registered by the dissolution of the distinct mitochondrial illumination pattern, which leads to a diffuse staining in the cytoplasm of these cells (Figures 5a and b).

### PepNoxa and pepBad do not individually induce apoptosis

**Figure 6:**
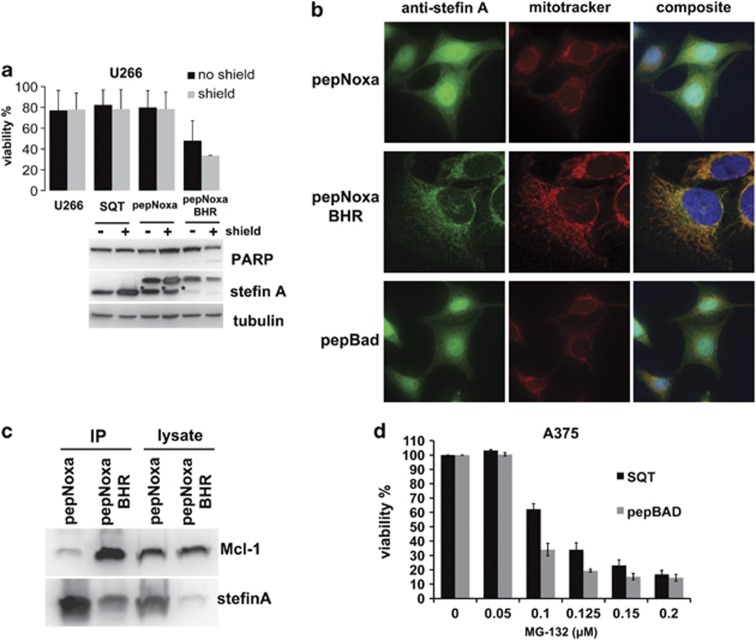
Apoptotic potential and cellular localisation of pepNoxa and pepBad. (**a**) Expression of pepNoxa in U266 cells has no effect on cell viability and does not cause PARP cleavage. PepNoxa with the BHR domain efficiently induces cell death and leads to PARP cleavage (error bars are ±S.D.; *n*=3). The inducible expression system is leaky in this cell type and thus pepNoxa-BHR expression occurs even in the absence of shield. The set of columns labelled ‘U266' denotes viability measurements of untransfected cells (which are not 100% viable). PepBH3 degradation products are indicated by an asterisk (*). (**b**) Immunofluorescence analysis of Hela cells expressing pepNoxa or pepBad (green) reveals no mitochondrial (red) localisation. PepNoxa-BHR colocalises very specifically with mitotracker (red). (**c**) Co-IP experiment using an anti-Stefin A antibody and lysate from cells expressing pepNoxa or pepNoxa-BHR. PepNoxa-BHR is expressed at lower levels, but purifies with a larger amount of Mcl-1 than pepNoxa lacking a BHR. (**d**) PepBad and MG-132 synergise to reduce viability in A375 cells (most prominently at 100 nM MG-132; error bars are ±S.D.; *n*=3)

Expression of pepNoxa or pepBad did not lead to cleavage of PARP in A375 cells (Figure 3), indicating that apoptosis was not induced by these anchored BH3 helices. A similar observation has been made with the full-length proteins, and was explained by the fact that Noxa and Bad neutralise only a subset of anti-apoptotic Bcl-2 proteins.^12^ To test pepNoxa's apoptotic potential, a U266 myeloma cell line was used, whose survival is dependent on Mcl-1.^30^ However, expression of pepNoxa in this cell line was not sufficient to drive apoptosis, as viability was unaffected and PARP was not cleaved (Figure 6a). It has previously been shown that the full-length Noxa protein possesses a C-terminal mitochondrial-targeting domain, whose presence is required for induction of apoptosis.^31^ In order to test whether targeting pepNoxa to the mitochondrion might result in induction of cell death, Bim's membrane-targeting domain (Bim hydrophobic region, BHR)^32^ was introduced into the C-terminus of pepNoxa. Expression of the pepNoxa-BHR protein in U266 cells led to a clear reduction in cell numbers compared with SQT or pepNoxa alone (Figure 6a). Fluorescent microscopy in Hela cells confirmed that pepNoxa-BHR localises to the mitochondria, in contrast to pepNoxa, which exhibits a diffuse staining pattern (Figure 6b). Co-IP using an anti-Stefin A antibody to capture pepNoxa revealed that inclusion of the BHR significantly increases the formation of the pepNoxa:Mcl-1 complex (Figure 6c). Finally, as pepBad is predicted to mimic the effects of the chemical Bcl-2 inhibitor ABT-737 and synergise with the proteasomal inhibitor MG-132,^33^ the ability of pepBad to promote apoptosis in A375 cells treated with MG-132 was tested (Figure 6d). The strongest effect was measured at a concentration of 100 nM of MG-132, where expression of pepBad leads to approximately 50% reduced cell viability compared with the empty SQT scaffold. As with full-length Bad, which has no known membrane-targeting sequence, pepBad's localisation pattern in Hela cells is diffused (Figure 6b).

## Discussion

The study of BH3 helices has value in basic research to help further elucidate the role of these domains in apoptosis and the regulation of mitochondrial fission, and in translational research to inform drug design. However, in some cases the BH3 helix alone is not sufficient to mediate biological function, and further tools are required. Here the use of the SQT peptide aptamer scaffold to present BH3 peptides has been successfully validated.

### At endogenous levels Bcl-2 does not interact with the BH3-only proteins

The binding of the BH3 helices to members of the Bcl-2 family has been thoroughly investigated over the past 10 years. Chen *et al.*^12^ have systematically tested the binding of several BH3 peptides to the five anti-apoptotic Bcl-2 proteins *in vitro*, as well as the interaction between full-length BH3-only proteins and a subset of the anti-apoptotic proteins following their overexpression in cells. Their findings match our observations to a large extent, with Puma and Bim binding to both endogenous Bcl-xL and Mcl-1 and Bad binding strongly to Bcl-xL but not Mcl-1 whereas Noxa was selective for Mcl-1. Interestingly, however, endogenous Bcl-2 did not co-IP with any of the pepBH3 (in A375 or Hela cells), or with endogenous, full-length Bad (in A375 or 293T cells). In the scientific literature there are, to our knowledge, only a few instances of endogenous Bcl-2 co-IP, and in those studies the cell types used are almost exclusively either mature^34^ or immature T cells^14, 35^ or B cells;^36^ all cell types which were not investigated in this study. The vast bulk of the literature has only reported positive BH3:Bcl-2 interactions *in vitro*^8, 12, 37^ or with overexpressed Bcl-2 inside cells.^12, 27, 38, 39^ The latter observation could be successfully reproduced in this study. Notable in this context is the fact that several haematological malignancies are driven by translocation of the *bcl-2* gene and consequent overexpression of the protein.^40^ Furthermore, there is some evidence that Bcl-2 is a much weaker pro-survival factor than, for instance, Bcl-xL^41^ and work with mouse embryonic fibroblasts has revealed that sequestration of Bcl-xL and Mcl-1 is sufficient to induce apoptosis.^42^ Thus, we hypothesise that, in most cell types, Bcl-2 might only be a relevant anti-apoptotic player and BH3-only protein inhibitor in the overexpressed state. Evidence corroborating our findings comes from Deng *et al.*^36^ who tested Bcl-2:Bim interaction via IP in 18 different diffuse large B-cell lymphoma cell lines. Importantly, they find that significant Bcl-2:Bim complex formation is generally only detectable in those cell lines carrying the t(14;18) translocation, where Bcl-2 levels are high. Recently, Plötz *et al.*^43^ co-IP BimL with endogenous Bcl-2 in Mel-2a melanoma cells, a cell line known to overexpress Bcl-2, particularly compared with A375, the melanoma cell line used in this study.^44^

### Only the anchored Bim BH3 helix interacts with Bax in A375 cells

The interaction of the BH3-only proteins with the anti-apoptotic Bcl-2 members serves to antagonise their function. There is, however, mounting evidence that Bax and Bak are directly activated by some of the BH3-only proteins.^15, 16^ In this study, only the anchored Bim *α*-helix was found to bind to Bax in cells. This observation is consistent with that by Mérino *et al.*^35^ who report binding of Bax to the Bim isoform Bim_S_. Replacement of the Bim BH3 peptide within the Bim backbone with that of Bad, Noxa or Puma led to no interaction. A different study, investigating binding to both Bax and Bak, found that Bim_S_ IP with Bax but found no interaction between Bak and the BH3-only proteins, consistent with our results.^45^ Both studies have also looked at the binding affinity between Bax and the Bim BH3 peptide biophysically, and only low (*μ*M) affinities were measured. Here we show that an anchored Bim BH3 peptide is sufficient to interact with Bax in cells. The efficiency of the co-IP, which is comparable to that of pepBim and Bcl-xL, suggests that the interaction *in vivo* is robust. Furthermore, Puma has previously been shown to promote oligomerisation and activation of Bax and Bak followed by cytochrome *c* release in isolated mitochondria^17^ and a bacterial two-hybrid assay has identified Puma as well as its BH3 peptide alone as interactors of Bax.^46^ In this study, however, no binding of the constrained Puma helix to either Bax or Bak was detected. Others using IP assays have also found that full-length Puma does not bind to the pro-apoptotic effector proteins in lysed cells.^45^

### The BH3 domain of Puma and Bim is sufficient to induce apoptosis; a membrane-targeting sequence is not required

One of the advantages of the anchored peptide system described here is that it allows the study of the BH3 peptides in a neutral and structurally stable scaffold outside the context of their native proteins. Previously, Weber *et al.*^29^ have shown that a truncated Bim_S_ lacking the C-terminal hydrophobic region was incapable of localising to the mitochondria and could not induce apoptosis. Equally a truncated Puma, with an intact BH3 domain but no C-terminus failed to target the mitochondria and kill cells.^28^ Here we show that the anchored BH3 helices of both Puma and Bim exhibit a strong apoptotic effect in A375 cells. In Hela cells, pepPuma powerfully induces cytochrome *c* release (pepBim was not tested). Although pepBim and pepPuma localise only poorly to the mitochondria, probably because the hydrophobic C-terminus is not present, these proteins do show increased accumulation around mitochondria compared with SQT alone. These findings thus prove that the Puma/Bim BH3 helix alone is enough to cause apoptosis even in the absence of efficient membrane targeting. In support of these results, several studies have successfully induced apoptosis in cancer cells by using a cell-permeable stapled BH3 peptides.^16, 47^ Furthermore, previous studies have shown that the Bim helix colocalises well – but not perfectly – with mitochondria,^47^ and that, following transfection of the Bim BH3 peptide into HeLa cells, cytochrome *c* release was observed in most cells.^11^

A further interesting observation reported here is that the levels of anti-apoptotic proteins fall approximately 2–4 h after induction of pepBim or pepPuma expression. The ability of the BH3-only proteins to regulate the stability of Mcl-1 has been investigated before, and targeting of the death antagonists to the proteasome by the pepBH3 proteins constitutes a potential explanation for these findings.^42, 48^

### Noxa requires a membrane-targeting domain to induce apoptosis; Bad does not

Contrary to Bim and Puma, which do not require their C-terminal hydrophobic region to cause cell death, the results obtained here show that Noxa has to be targeted to intracellular membranes to do so. The successful use of the BHR to target pepNoxa to the mitochondria, instead of Noxa's own mitochondrial-targeting domain,^31^ shows that, to induce cell death, Noxa does not require a specific targeting sequence, but simply needs to localise to the mitochondria. This has potential implications for the design of Mcl-1 (Noxa's target) inhibitors to treat malignancies such as chronic lymphocytic leukaemia and multiple myeloma, which have been shown to be dependent on Mcl-1 for survival.^49, 50^ PepBad, on the other hand, showed apoptotic potential in the absence of a membrane-targeting domain consistent with the fact that the natural full-length protein lacks such a feature.^51^ Consequently, the results support the idea that the potency of the BH3-only Bcl-2 proteins is not only determined by their affinity for the anti-apoptotic proteins, but is also regulated by their intracellular localisation.

### Conclusion

The anchored helix approach to studying the BH3 peptides constitutes a way of informing the growing field of potential therapeutics that are the BH3 helix mimetics^18, 19, 52, 53, 54^ and stapled helices,^37, 55, 56^ as it presents a neutral and robust platform to study the intracellular effects of the BH3 domains outside the context of the native protein backbone. Other methods, such as using Bim_S_ as a common backbone to study the BH3 helices by replacing the Bim BH3 sequence with that of the BH3-only protein to be studied have been used successfully in the past.^12, 35^ This approach, however, might not always reflect the true character of the BH3 domain under investigation as the BHR targets the protein to intracellular membranes very efficiently. Another important finding reported here is the apparent inability of Bcl-2 to interact with the pro-apoptotic BH3-only proteins, when the former is only present at endogenous levels in the cell. Conversely, when overexpressed, binding occurs readily. This suggests that Bcl-2's role in counteracting apoptosis might only be significant in specific pathological situations where the protein's expression levels are unnaturally high (e.g., following genetic translocation). Future studies should prioritise the verification of this hypothesis.

## Materials and Methods

### DNA manipulation and cloning

Double-stranded DNA cassettes encoding each BH3 peptide (Figure 1a) or the BHR flanked by the *Avr*II or *Xho*I restriction enzyme site, respectively, were made by annealing oligonucleotides (Supplementary Table S1). For bacterial expression, restriction enzyme digested dsDNA cassettes were ligated into the appropriate restriction sites of the SQT scaffold-encoding ORF in pET30a(+).^23^ For viral transfection, each construct was amplified by polymerase chain reaction (PCR) from pET30a(+) using SQT-specific primers (F 5′-CCGCGGCCGCAGATCTTCATGATACCTAGG-3′, R 5′-GAGAGGGGCGCCATGCTAAAAGCCCGTCAGCTCG-3′). PCR products were cloned into the Retro-X ProteoTuner vector using In-Fusion cloning and sequenced, as per manufacturer's instructions (Clontech, St-Germain-en-Laye, France).

### PepBH3 expression in *E. coli* and purification

pET30a(+) expression vectors containing the pepBH3 ORF were transformed into BL21 (DE3) pLysS competent cells (Stratagene, Santa Clara, CA, USA) and expressed under induction with IPTG for 3 h at 37 °C. Cells were harvested, lysed using a sonicator, and the recombinant protein purified using Ni-NTA sepharose beads (Novagen, Billerica, MA, USA), as described previously.^57^

### Production of retrovirus and transduction of cells

Constructs were transfected into Phoenix A packaging cells (ATCC, Manassas, VA, USA), using siPORT XP-1 transfection agent (Life Technologies, Carlsbad, CA, USA). After 48 h, the medium was harvested, 0.4 *μ*m filtered and mixed in equal amounts with fresh medium containing 8 *μ*g/ml of polybrene (Sigma, Gillingham, UK). Cells were incubated with retroviral supernatants for 8 h. At 48 h after transduction, cells were transferred into selection medium containing puromycin.

### Transient transfection of A375 cells

A pCMV-Tag2B vector carrying Bcl-2 with an N-terminal Flag-tag was ordered from Addgene (Cambridge, MA, USA). The transfection mix was assembled as follows: 3.3 *μ*g of high-quality plasmid DNA (midiprep) per six-well plate well is diluted in sterile water to a concentration of 20 ng/*μ*l. Then FuGeneHD transfection reagent (Promega, Southampton UK), which has been warmed to room temperature (RT), is added directly to the solution at 10 *μ*l/3.3 *μ*g DNA. This is followed by brief vortexing and incubation at RT for 5–10 min. The mix is then added to cells, which are approximately 80% confluent, and are grown in complete DMEM GlutaMAX medium (Life Technologies, Paisley, UK), with 3 ml of the medium per six-well plate well. The transfected cells were harvested after 24 h for downstream applications.

### Far-UV CD spectroscopy

CD spectra were measured on a Jasco J715 spectropolarimeter (Jasco, Easton, MD, USA) as described previously.^57^ Briefly, pepBH3 proteins were transferred into 50 mM sodium phosphate buffer (pH 7.4) and their CD spectra recorded from 200 to 260 nm. The spectrum for buffer alone was substracted from the measurements and the data analysed and visualised using Microsoft Excel.

### IP experiments

IP experiments were carried out using the Crosslinking Immunoprecipitation kit (Pierce, Rockford, IL, USA) according to the manufacturer's instructions. Briefly, cells expressing the desired protein were lysed using the kit's IP lysis/wash buffer (containing a non-ionic detergent) and the soluble fraction applied to agarose beads carrying the appropriate antibody (*α*-Bad, Abcam (Cambridge, UK); *α*-Stefin A, R&D Systems (Minneapolis, MN, USA); *α*-Flag-Tag, Sigma). Lysates were incubated overnight at 4 °C and the beads were then washed four times and eluted with a low-pH buffer. Elution fractions were stored at −80 °C until analysis by western blotting.

### Western blotting

Western blotting was preceded by sodium dodecyl sulphate polyacrylamide gel electrophoresis and transfer onto nitrocellulose or PVDF membrane. The membrane was blocked in 3% milk for 1 h at RT followed by incubation with antibody at 4 °C overnight. Antibodies used were *α*-Stefin A (1 : 3000; R&D Systems), *α*-tubulin (1 : 10 000; AbD Serotec, Kidlington, UK), *α*s-Mcl-1, *α*-Bcl-2, *α*-Bcl-xL, *α*-Bad, *α*-Bak and *α*-Bax (all 1 : 1000; all from Cell Signalling Technology, Hitchin, UK) and *α*-PARP (1 : 1000; BD Biosciences, Oxford, UK). HRP-conjugated secondary antibodies were *α*-mouse, *α*-rabbit, *α*-goat (all 1 : 12 000; all from Jackson Immunoresearch, West Grove, PA, USA) and were applied for 1 h at RT. For detection, the membrane was incubated with ECL reagent (Pierce) and exposed to photographic film.

### Immunocytochemistry

For ICC, Hela cells were cultured on glass cover slips. Mitotracker red CMXRos (25 nM; Life Technologies, Carlsbad, CA, USA) was added to the culture medium 30 min before fixing in 4% paraformaldehyde. Cells were permeabilised in 2% Triton-X100 for 5 min, washed in PBS and blocked in 2% milk for 30 min. Subsequently, the anti-Stefin A antibody was applied (1 : 100; R&D Systems) for 2 h at RT followed by incubation with Alexa Fluor 488 labelled secondary antibody (1 : 200; Life Technologies, Carlsbad, CA, USA) and 4',6-diamidino-2-phenylindole (DAPI) for 1 h. Before fluorescent microscopy, the cover slips were mounted onto glass slides using ProLong Gold antifade reagent (Life Technologies, Carlsbad, CA, USA).

### Monitoring cell viability and cytochrome *c* release

Viability and apoptosis assays were performed using the Guava EasyCyte System (Millipore, Billerica, MA, USA), according to the manufacturer's instructions. Hela cells, which stably express GFP–cytochrome *c* (a kind gift from Douglas Green, St. Jude Children's Research Hospital, Memphis, TN, USA), were transfected with pepPuma as described above. One hour after addition of Shield, the cells were monitored under green fluorescence and micrographs taken every 5 min for 2 h.

## Abbreviations

A1: Bcl-2-related gene A1
Bad: Bcl-2-associated death promoter
Bak: Bcl-2 antagonist killer
Bax: Bcl-2-associated x protein
Bcl-2: B-cell CLL/lymphoma-2
Bcl-xL: Bcl-2-related gene long isoform
BH domain: Bcl-2 homology domain
Bid: Bcl-2-interacting domain death agonist
Bik: Bcl-2-interacting killer
Bim: Bcl-2-interacting mediator of cell death
CD: circular dichroism
DAPI: 4',6-diamidino-2-phenylindole
GFP: green fluorescent protein
MOM(P): mitochondrial outer membrane (permeabilisation)
PCR: polymerase chain reaction
Puma: p53 upregulated modulator of apoptosis
SQT: Stefin A quadruple mutant Tracy

## References

[bib1] Adams JM Cory S Bcl-2-regulated apoptosis: mechanism and therapeutic potential Curr Opin Immunol 2007 19 488 496 17629468 10.1016/j.coi.2007.05.004 PMC2754308

[bib2] Brunelle JK Letai A Control of mitochondrial apoptosis by the Bcl-2 family J Cell Sci 2009 122 437 441 19193868 10.1242/jcs.031682 PMC2714431

[bib3] Chipuk JE Moldoveanu T Llambi F Parsons MJ Green DR The BCL-2 family reunion Mol Cell 2010 37 299 310 20159550 10.1016/j.molcel.2010.01.025 PMC3222298

[bib4] Happo L Strasser A Cory S BH3-only proteins in apoptosis at a glance J Cell Sci 2012 125 (Pt 5 1081 1087 22492984 10.1242/jcs.090514 PMC3324577

[bib5] Shamas-Din A Kale J Leber B Andrews DW Mechanisms of action of Bcl-2 family proteins Cold Spring Harb Perspect Biol 2013 5 a008714 23545417 10.1101/cshperspect.a008714 PMC3683897

[bib6] Brooks C Dong Z Regulation of mitochondrial morphological dynamics during apoptosis by Bcl-2 family proteins: a key in Bak Cell Cycle 2007 6 3043 3047 18073534 10.4161/cc.6.24.5115

[bib7] Germain M Slack RS Dining in with BCL-2: new guests at the autophagy table Clin Sci (Lond) 2009 118 173 181 19845510 10.1042/CS20090310

[bib8] Letai A Bassik MC Walensky LD Sorcinelli MD Weiler S Korsmeyer SJ Distinct BH3 domains either sensitize or activate mitochondrial apoptosis, serving as prototype cancer therapeutics Cancer Cell 2002 2 183 192 12242151 10.1016/s1535-6108(02)00127-7

[bib9] Moreau C Cartron P-F Hunt A Meflah K Green DR Evan G Minimal BH3 peptides promote cell death by antagonizing anti-apoptotic proteins J Biol Chem 2003 278 19426 19435 12642586 10.1074/jbc.M209472200

[bib10] Villunger A Michalak E Coultas L Müllauer F Böck G Ausserlechner MJ p53- and drug-induced apoptotic responses mediated by BH3-only proteins puma and noxa Science 2003 302 1036 1038 14500851 10.1126/science.1090072

[bib11] Kuwana T Bouchier-Hayes L Chipuk JE Bonzon C Sullivan BA Green DR BH3 domains of BH3-only proteins differentially regulate Bax-mediated mitochondrial membrane permeabilization both directly and indirectly Mol Cell 2005 17 525 535 15721256 10.1016/j.molcel.2005.02.003

[bib12] Chen L Willis SN Wei A Smith BJ Fletcher JI Hinds MG Differential targeting of prosurvival Bcl-2 proteins by their BH3-only ligands allows complementary apoptotic function Mol Cell 2005 17 393 403 15694340 10.1016/j.molcel.2004.12.030

[bib13] Certo M Del Gaizo Moore V Nishino M Wei G Korsmeyer S Armstrong SA Mitochondria primed by death signals determine cellular addiction to antiapoptotic BCL-2 family members Cancer Cell 2006 9 351 365 16697956 10.1016/j.ccr.2006.03.027

[bib14] Opferman JT Letai A Beard C Sorcinelli MD Ong CC Korsmeyer SJ Development and maintenance of B and T lymphocytes requires antiapoptotic MCL-1 Nature 2003 426 671 676 14668867 10.1038/nature02067

[bib15] Kuwana T Mackey MR Perkins G Ellisman MH Latterich M Schneiter R Bid, Bax, and lipids cooperate to form supramolecular openings in the outer mitochondrial membrane Cell 2002 111 331 342 12419244 10.1016/s0092-8674(02)01036-x

[bib16] Gavathiotis E Suzuki M Davis ML Pitter K Bird GH Katz SG BAX activation is initiated at a novel interaction site Nature 2008 455 1076 1081 18948948 10.1038/nature07396 PMC2597110

[bib17] Kim H Rafiuddin-Shah M Tu H-C Jeffers JR Zambetti GP Hsieh JJ-D Hierarchical regulation of mitochondrion-dependent apoptosis by BCL-2 subfamilies Nat Cell Biol 2006 8 1348 1358 17115033 10.1038/ncb1499

[bib18] Oltersdorf T Elmore SW Shoemaker AR Armstrong RC Augeri DJ Belli BA An inhibitor of Bcl-2 family proteins induces regression of solid tumours Nature 2005 435 677 681 15902208 10.1038/nature03579

[bib19] Labi V Grespi F Baumgartner F Villunger A Targeting the Bcl-2-regulated apoptosis pathway by BH3 mimetics: a breakthrough in anticancer therapy Cell Death Differ 2008 15 977 987 18369371 10.1038/cdd.2008.37 PMC4563920

[bib20] Adessi C Soto C Converting a peptide into a drug: strategies to improve stability and bioavailability Curr Med Chem 2002 9 963 978 11966456 10.2174/0929867024606731

[bib21] Sadowsky JD Murray JK Tomita Y Gellman SH Exploration of backbone space in foldamers containing alpha- and beta-amino acid residues: developing protease-resistant oligomers that bind tightly to the BH3-recognition cleft of Bcl-xL Chembiochem 2007 8 903 916 17503422 10.1002/cbic.200600546

[bib22] Hamacher K Hübsch A McCammon JA A minimal model for stabilization of biomolecules by hydrocarbon cross-linking J Chem Phys 2006 124 164907 164914 16674170 10.1063/1.2185645

[bib23] Stadler LK Hoffmann T Tomlinson DC Song Q Lee T Busby M Structure-function studies of an engineered scaffold protein derived from Stefin A. II: Development and applications of the SQT variant Protein Engineering Design Selection 2011 24 751 763 10.1093/protein/gzr019 21616931

[bib24] Colas P Cohen B Jessen T Grishina I McCoy J Brent R Genetic selection of peptide aptamers that recognize and inhibit cyclin-dependent kinase 2 Nature 1996 380 548 550 8606778 10.1038/380548a0

[bib25] Wilson-Annan J O'Reilly LA Crawford SA Hausmann G Beaumont JG Parma LP Proapoptotic BH3-only proteins trigger membrane integration of prosurvival Bcl-w and neutralize its activity J Cell Biol 2003 162 877 887 12952938 10.1083/jcb.200302144 PMC2172834

[bib26] Banaszynski LA Chen LC Maynard-Smith LA Ooi AG Wandless TJ A rapid, reversible, and tunable method to regulate protein function in living cells using synthetic small molecules Cell 2006 126 995 1004 16959577 10.1016/j.cell.2006.07.025 PMC3290523

[bib27] Nakano K Vousden KH PUMA, a novel proapoptotic gene, is induced by p53 Mol Cell 2001 7 683 694 11463392 10.1016/s1097-2765(01)00214-3

[bib28] Yu J Wang Z Kinzler KW Vogelstein B Zhang L PUMA mediates the apoptotic response to p53 in colorectal cancer cells Proc Natl Acad Sci USA 2003 100 1931 1936 12574499 10.1073/pnas.2627984100 PMC149936

[bib29] Weber A Paschen SA Heger K Wilfling F Frankenberg T Bauerschmitt H BimS-induced apoptosis requires mitochondrial localization but not interaction with anti-apoptotic Bcl-2 proteins J Cell Biol 2007 177 625 636 17517961 10.1083/jcb.200610148 PMC2064208

[bib30] Derenne S Monia B Dean NM Taylor JK Rapp M-J Harousseau J-L Antisense strategy shows that Mcl-1 rather than Bcl-2 or Bcl-x(L) is an essential survival protein of human myeloma cells Blood 2002 100 194 199 12070027 10.1182/blood.v100.1.194

[bib31] Seo Y-W Shin JN Ko KH Cha JH Park JY Lee BR The molecular mechanism of Noxa-induced mitochondrial dysfunction in p53-mediated cell death J Biol Chem 2003 278 48292 48299 14500711 10.1074/jbc.M308785200

[bib32] O'Connor L Strasser A O'Reilly LA Hausmann G Adams JM Cory S Bim: a novel member of the Bcl-2 family that promotes apoptosis The EMBO J 1998 17 384 395 10.1093/emboj/17.2.384 PMC1170389 9430630

[bib33] Miller LA Goldstein NB Johannes WU Walton CH Fujita M Norris DA BH3 mimetic ABT-737 and a proteasome inhibitor synergistically kill melanomas through Noxa-dependent apoptosis J Invest Dermatol 2009 129 964 971 18987671 10.1038/jid.2008.327

[bib34] Zhu Y Swanson BJ Wang M Hildeman DA Schaefer BC Liu X Constitutive association of the proapoptotic protein Bim with Bcl-2-related proteins on mitochondria in T cells Proc Natl Acad Sci USA 2004 101 7681 7686 15136728 10.1073/pnas.0402293101 PMC419666

[bib35] Mérino D Giam M Hughes PD Siggs OM Heger K O'Reilly LA The role of BH3-only protein Bim extends beyond inhibiting Bcl-2-like prosurvival proteins J Cell Biol 2009 186 355 362 19651893 10.1083/jcb.200905153 PMC2728397

[bib36] Deng J Carlson N Takeyama K Dal Cin P Shipp M Letai A BH3 profiling identifies three distinct classes of apoptotic blocks to predict response to ABT-737 and conventional chemotherapeutic agents Cancer Cell 2007 12 171 185 17692808 10.1016/j.ccr.2007.07.001

[bib37] Walensky LD Kung AL Escher I Malia TJ Barbuto S Wright RD Activation of apoptosis *in vivo* by a hydrocarbon-stapled BH3 helix Science 2004 305 1466 1470 15353804 10.1126/science.1099191 PMC1360987

[bib38] Llambi F Moldoveanu T Tait SW Bouchier-Hayes L Temirov J McCormick LL A unified model of mammalian BCL-2 protein family interactions at the mitochondria Mol Cell 2011 44 517 531 22036586 10.1016/j.molcel.2011.10.001 PMC3221787

[bib39] Aranovich A Liu Q Collins T Geng F Dixit S Leber B Differences in the mechanisms of proapoptotic BH3 proteins binding to Bcl-XL and Bcl-2 quantified in live MCF-7 cells Mol Cell 2012 45 754 763 22464442 10.1016/j.molcel.2012.01.030

[bib40] Galteland E Sivertsen EA Svendsrud DH Smedshammer L Kresse SH Meza-Zepeda LA Translocation t(14;18) and gain of chromosome 18/BCL2: effects on BCL2 expression and apoptosis in B-cell non-Hodgkin's lymphomas Leukemia 2005 19 2313 2323 16193090 10.1038/sj.leu.2403954

[bib41] Fiebig AA Zhu W Hollerbach C Leber B Andrews DW Bcl-XL is qualitatively different from and ten times more effective than Bcl-2 when expressed in a breast cancer cell line BMC Cancer 2006 6 213 16928273 10.1186/1471-2407-6-213 PMC1560389

[bib42] Willis SN Chen L Dewson G Wei A Naik E Fletcher JI Proapoptotic Bak is sequestered by Mcl-1 and Bcl-xL, but not Bcl-2, until displaced by BH3-only proteins Genes Dev 2005 19 1294 1305 15901672 10.1101/gad.1304105 PMC1142553

[bib43] Plötz M Gillissen B Quast S-A Berger A Daniel PT Eberle J The BH3-only protein BimL overrides Bcl-2-mediated apoptosis resistance in melanoma cells Cancer Lett 2013 335 100 108 23402819 10.1016/j.canlet.2013.02.005

[bib44] Bektas M Jolly PS Müller C Eberle J Spiegel S Geilen CC Sphingosine kinase activity counteracts ceramide-mediated cell death in human melanoma cells: role of Bcl-2 expression Oncogene 2005 24 178 187 15637591 10.1038/sj.onc.1208019

[bib45] Willis SN Fletcher JI Kaufmann T van Delft MF Chen L Czabotar PE Apoptosis initiated when BH3 ligands engage multiple Bcl-2 homologs, not Bax or Bak Science 2007 315 856 859 17289999 10.1126/science.1133289

[bib46] Cartron P-F Gallenne T Bougras G Gautier F Manero F Vusio P The first alpha helix of Bax plays a necessary role in its ligand-induced activation by the BH3-only proteins Bid and PUMA Mol Cell 2004 16 807 818 15574335 10.1016/j.molcel.2004.10.028

[bib47] LaBelle JL Katz SG Bird GH Gavathiotis E Stewart ML Lawrence C A stapled BIM peptide overcomes apoptotic resistance in hematologic cancers J Clin Invest 2012 122 2018 2031 22622039 10.1172/JCI46231 PMC3366394

[bib48] Czabotar PE Lee EF van Delft MF Day CL Smith BJ Huang DCS Structural insights into the degradation of Mcl-1 induced by BH3 domains Proc Natl Acad Sci USA 2007 104 6217 6222 17389404 10.1073/pnas.0701297104 PMC1851040

[bib49] Zhang B Gojo I Fenton RG Myeloid cell factor-1 is a critical survival factor for multiple myeloma Blood 2002 99 1885 1893 11877256 10.1182/blood.v99.6.1885

[bib50] Alvi AJ Austen B Weston VJ Fegan C MacCallum D Gianella-Borradori A A novel CDK inhibitor, CYC202 (R-roscovitine), overcomes the defect in p53-dependent apoptosis in B-CLL by down-regulation of genes involved in transcription regulation and survival Blood 2005 105 4484 4491 15692065 10.1182/blood-2004-07-2713

[bib51] Lomonosova E Chinnadurai G BH3-only proteins in apoptosis and beyond: an overview Oncogene 2008 27 (Suppl 1 S2 S19 19641503 10.1038/onc.2009.39 PMC2928556

[bib52] Carrington EM Vikstrom IB Light A Sutherland RM Londrigan SL Mason KD BH3 mimetics antagonizing restricted prosurvival Bcl-2 proteins represent another class of selective immune modulatory drugs Proc Natl Acad Sci USA 2010 107 10967 10971 20534453 10.1073/pnas.1005256107 PMC2890751

[bib53] Zhang Z Song T Zhang T Gao J Wu G An L A novel BH3 mimetic S1 potently induces Bax/Bak-dependent apoptosis by targeting both Bcl-2 and Mcl-1 Int J Cancer 2010 128 1724 1735 20503275 10.1002/ijc.25484

[bib54] Kazi A Sun J Doi K Sung SS Takahashi Y Yin H The BH3 alpha-helical mimic BH3-M6 disrupts Bcl-X(L), Bcl-2, and MCL-1 protein-protein interactions with Bax, Bak, Bad, or Bim and induces apoptosis in a Bax- and Bim-dependent manner J Biol Chem 2011 286 9382 9392 21148306 10.1074/jbc.M110.203638 PMC3059047

[bib55] Walensky LD Pitter K Morash J Oh KJ Barbuto S Fisher J A stapled BID BH3 helix directly binds and activates BAX Mol Cell 2006 24 199 210 17052454 10.1016/j.molcel.2006.08.020

[bib56] Stewart ML Fire E Keating AE Walensky LD The MCL-1 BH3 helix is an exclusive MCL-1 inhibitor and apoptosis sensitizer Nat Chem Biol 2010 6 595 601 20562877 10.1038/nchembio.391 PMC3033224

[bib57] Hoffmann T Stadler LKJ Busby M Song Q Buxton AT Wagner SD Structure-function studies of an engineered scaffold protein derived from Stefin A. I: development of the SQM variant Prot Eng Des Sel 2010 23 403 413 10.1093/protein/gzq012 PMC2851446 20179045

