# Supplementary material for: The use of a neutral peptide aptamer scaffold to anchor BH3 peptides constitutes a viable approach to studying their function
Source: Cell Death Dis. 2014 Jan 30;5(1):e1037–. doi: 10.1038/cddis.2013.564 (PMC4040713; doi:10.1038/cddis.2013.564)
Supplement: Supplementary Table S1 [file cddis2013564x1.doc]

| primer name | orientation | nucleotide sequence (5’-3’) |
| --- | --- | --- |
| Puma_F | fwd | GATACCTAGGGAGGAGCAATGGGCTCGTGAGATTGGTGCTCAACTGCGTCGTATGGC |
| Puma_R | rev | AGCCCCTAGGACGACGTTCATATTGAGCATTCAGATCATCAGCCATACGACGCAGTTG |
| Bad_F | fwd | GATACCTAGGAACTTATGGGCTGCCCAACGTTATGGTAGAGAATTGCGTCGCATGTC |
| Bad_R | rev | AGCCCCTAGGGCCTTTCTTGAAGGAATCGACAAACTCATCAGACATGCGACGCAATTC |
| Bim_F | fwd | GATACCTAGGGATATGCGTCCTGAAATTTGGATTGCTCAAGAACTGCGTCGTATTGG |
| Bim_R | rev | GATACCTAGGACGACGAGCATAATAAGCATTGAATTCATCACCAATACGACGCAGTTC |
| Noxa_F | fwd | GATACCTAGGCCTGCTGAATTAGAGGTTGAATGTGCTACACAACTCAGAAGATTTGG |
| Noxa_R | rev | GATACCTAGGGAGAAGCTTTTGTCTAAAATTAAGTTTATCGCCAAATCTTCTGAGTTG |
| Puma_delF | fwd | GATACCTAGGGAGGAGCAATGGGCTCGTGAGATTGGTGCTCAAATGGCTGATG |
| Puma_delR | rev | AGCCCCTAGGACGACGTTCATATTGAGCATTCAGATCATCAGCCATTTGAGCAC |
| BHR_F | fwd | TCGAGATGGTTATTCTTCAACTTCTTCGTTTTATTTTCCGCTTGGTGTGGC |
| BHR_R | rev | TCGAGCCACACCAAGCGGAAAATAAAACGAAGAAGTTGAAGAATAACCATC |

**Table S1. Oligonucleotide sequences**
